# An update on the phylogeny of capillariid nematodes based on 18S rDNA sequences of *Amphibiocapillaria tritonispunctati* (Diesing, 1851) and four other species

**DOI:** 10.1016/j.crpvbd.2025.100321

**Published:** 2025-09-19

**Authors:** Roman Svitin, Yaroslav Syrota, Yuriy Kuzmin, Valeriia Dupak, Oksana Nekrasova, Oleksii Marushchak, Kateryna Antipova, Oksana Greben, Zuzana Hurníková, Nataliia Brusentsova, Martina Miterpáková

**Affiliations:** a I. I. Schmalhausen Institute of Zoology of National Academy of Sciences of Ukraine, B. Khmelnytskogo 15, 01054, Kyiv, Ukraine; b African Amphibian Conservation Research Group, Unit for Environmental Sciences and Management, North-West University, Potchefstroom Campus, Private Bag X6001, Potchefstroom, 20520, South Africa; c Educational and Scientific Institute of High Technologies of Taras Shevchenko National University of Kyiv, 60 Volodymyrska Street, Kyiv, 01033, Ukraine; d Université de Strasbourg, CNRS, IPHC UMR 7178, F-67000, Strasbourg, France; e Department of Ecology, Institute of Life Sciences and Technologies, Daugavpils University, LV5400, Daugavpils, Latvia; f Institute of Parasitology, Slovak Academy of Sciences, Hlinkova 3, 040 01, Košice, Slovakia; g Kuialnytskyi National Nature Park, Polska 21, 65014, Odesa, Ukraine

**Keywords:** Capillariidae, Phylogenetics, Parasite, Helminth, Nematoda, Europe, Amphibians

## Abstract

The nematode family Capillariidae represents a taxonomically complex and understudied group of parasitic nematodes infecting a broad range of vertebrate hosts. Despite more than 300 described species, phylogenetic relationships within the family remain unresolved due to limited molecular data and ongoing taxonomic revisions. In this study, we generated new sequences of the 18S ribosomal RNA gene for *Amphibiocapillaria tritonispunctati* from the Danube crested newt, *Triturus dobrogicus*, and four additional capillariid species (*Aonchotheca annulosa*, *Baruscapillaria inflexa*, *Eucoleus* sp. 1 from the common starling *Sturnus vulgaris*, and *Eucoleus* sp. 2 from the black-headed gull *Chroicocephalus ridibundus*) from birds and rodents, expanding the molecular dataset for the group. Phylogenetic analyses using Bayesian inference and Maximum Likelihood methods revealed *A. tritonispunctati* as the earliest-diverging lineage within the Capillariidae, suggesting deep evolutionary divergence. Our results also supported the monophyly of *Eucoleus* and *Capillaria* and confirmed the distinctness of *Baruscapillaria*. Morphological examination of *A. tritonispunctati* corroborated its identification and highlighted the weight of diagnostic characters of the genus *Amphibiocapillaria*. Our findings underscore the need for broader molecular sampling and integrative taxonomy to clarify capillariid systematics and host-parasite relationships, particularly among nematodes from cold-blooded vertebrates.

## Highlights

• Morphological description provided for *Amphibiocapillaria tritonispunctati.*
• Novel 18S rDNA sequences generated for *A. tritonispunctati*, *Aonchotheca annulosa*, *Baruscapillaria inflexa* and *Eucoleus* spp.
• Bayesian inference and maximum likelihood phylogenies recovered *A. ritonispunctati* as a basal taxon of the Capillariidae.

## Graphical abstract

**Figure:**
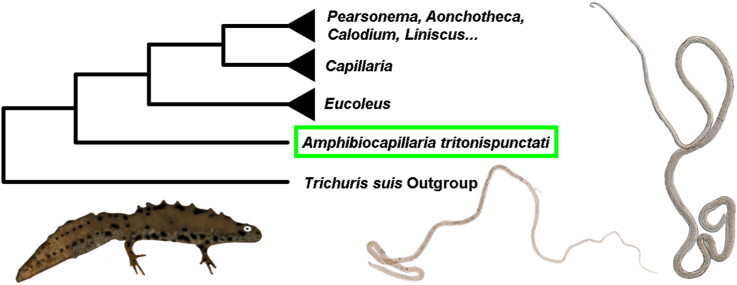

## 1 Introduction

Nematodes of the family Capillariidae comprise a diverse group of parasitic nematodes, with over 300 species currently described (Deng et al., 2021). These species are found in various hosts, including fishes, amphibians, reptiles, birds, and mammals worldwide, with new species being frequently described (de Carvalho et al., 2023; Charoennitiwat et al., 2024). The taxonomy within the family has been debated over the years, with several genera described, synonymised, reinstated, and divided into subgenera (Yamaguti, 1961; Moravec, 1982, 2001). Although molecular phylogenetic studies, mostly based on a partial fragment of the nuclear 18S ribosomal RNA gene (rDNA), have shed some light on the systematics of the Capillariidae, the resulting phylogenies still yield controversial inferences (Tamaru et al., 2015, 2025; Borba et al., 2019; Deng et al., 2021; Charoennitiwat et al., 2024). Most trees revealed two well-supported monophyletic clades formed by *Eucoleus* spp. and several species of *Capillaria* Zeder (1800), while species of *Pearsonema* Freitas & Mendonca, 1960, *Aonchotheca* Lopez-Neyra, 1947, *Pseudocapillaria* Freitas, 1959, *Baruscapillaria* Moravec, 1982; *Paratrichosoma* Ashford & Muller, 1978 appeared paraphyletic or clustered in poorly-supported clades (Borba et al., 2019; Sakaguchi et al., 2020; Svitin et al., 2021; Deng et al., 2021). Despite more than 70 nominal capillariid species described from cold-blooded hosts (Moravec, 2001; Charoennitiwat et al., 2024), only a few species have been included in the phylogenetic analyses to date, i.e. one species of *Pseudocapillaria* from the lungfish and two species of *Paracapillaria* Mendonca, 1963 from snakes (Svitin et al., 2021; Charoennitiwat et al., 2024). Furthermore, mitogenomes have been sequenced for only four genera, *Capillaria*, *Pseudocapillaria*, *Eucoleus* Dujardin, 1845, and *Aonchotheca*, and these data have been used to confirm that *Aonchotheca* is distinct from *Capillaria* (see Deng et al., 2021). Nonetheless, the number of available sequences remains limited, and despite the growing use of molecular methods, the small number of species still hinders the ability to draw robust conclusions about the taxonomic status and relationships among various capillariids.

In the present study, we aimed to clarify the phylogenetic position of *Amphibiocapillaria tritonispunctati* (Diesing, 1851) within the family Capillariidae. While the nematode’s distinctive morphology has historically posed taxonomic challenges (Moravec, 1982), its phylogenetic placement has remained unresolved by molecular methods. To address this, we utilised newly generated 18S rDNA sequences for *A. tritonispunctati* and four other capillariid species (*Aonchotheca annulosa*, *Baruscapillaria inflexa*, *Eucoleus* sp. 1, and *Eucoleus* sp. 2) to broaden the existing molecular dataset for the family.

## 2 Materials and methods

Most samples used in the study originated from Ukraine and were sourced from the collection of the Department of Parasitology of the I. I. Schmalhausen Institute of Zoology, National Academy of Sciences of Ukraine. Specimens of *A. tritonispunctati* were collected from Danube crested newts, *Triturus dobrogicus* Kiritzescu. These newts perished during the ecocide resulting from the destruction of the Kakhovka Dam on 6 June 2023. They were found dead on 13–15 June and collected on the Black Sea coast in Tuzlivski Lymany National Nature Park (Odesa Region), having been washed ashore by the devastating waters. A sample of 15 newts was dissected for parasitological studies. Specimens of *Eucoleus* sp. 1 and *Baruscapillaria inflexa* (Rudolphi, 1819; Travassos, 1915 were recovered from six common starlings, *Sturnus vulgaris* L., collected in the city of Hostomel (outskirts of Kyiv) in 2017. A specimen of *Eucoleus* sp. 2 was collected from a black-headed gull, *Chroicocephalus ridibundus* (L.), in Chernihiv Region in 2016. Additionally, two specimens of *Aonchotheca annulosa* (Dujardin, 1845; López-Neyra, 1947 were collected from two bank voles, *Myodes glareolus* (Schreber), in the Slovak Republic in 2023: one from Košice Zoo and another from the vicinity of the village Rozhanovce.

All helminth specimens were collected from thawed host carcasses, fixed in 70% ethanol, and stored in collections. For the morphological identification, nematodes were washed in distilled water for approximately 30 min, cleared in lactophenol or glycerol, and examined as temporary mounts in the same medium. Morphological identifications and photomicrographs were performed using light microscopes (ZEISS Axio Imager M1 and AmScope T690B). All measurements are given in micrometres unless otherwise indicated and presented as a range.

Genomic DNA (gDNA) was extracted from either whole nematode bodies or mid-body fragments (prior to lactophenol clearing) using the FavorPrep™ Tissue Genomic DNA Extraction Kit (Favorgen Biotech Corporation, Pingtung, Taiwan) following the manufacturer’s standard protocol. Each polymerase chain reaction (PCR) sample contained 2.0 μl of gDNA, 12.5 μl of MyTaq™ HS Red Mix (Bioline Reagents, London, UK), 1.0 μl of each primer (10 μM stock concentration), and 5.5 μl of nuclease-free water, in a total volume of 22 μl. A fragment (∼1500 bp) of the 18S rRNA gene was amplified using the primers NSF4/18 (5′-CTG GTT GAT CCT GCC AGT-3′) and NSR1787/18 (5′-CGA CGG GCG GTG TGT ACA-3′) (Harel et al., 2008). The thermocycling profile consisted of an initial denaturation at 94 °C for 2 min, followed by 30 cycles of 30 s at 94 °C, 30 s at 45 °C, and 1 min at 72 °C, with a final extension step of 7 min at 72 °C. In addition, for *A. tritonispunctati*, a ∼500 bp fragment of the mitochondrial cytochrome *c* oxidase subunit 1 (*cox*1) gene was amplified using the primers XWCOX1F (5′-ATY GGT GGA TTY GGW AAT TG-3′) and XWCOX1R (5′-TAA ACC TCN GGG TGH CCA A-3′) (Zou et al., 2017). The PCR protocol consisted of an initial denaturation at 94 °C for 2 min, followed by 30 cycles of 30 s at 94 °C, 30 s at 55 °C, and 30 s at 72 °C, with a final extension step of 7 min at 72 °C. PCR products were checked by electrophoresis on agarose gels alongside a DNA ladder. Amplicons were purified using the GeneAll® Expin™ Combo GP kit (GeneAll Biotechnology Co., Ltd., Seoul, South Korea). Sanger sequencing was performed by the commercial provider SEQme s.r.o. (Czech Republic) using the PCR primers. Resulting chromatograms were assembled and edited into consensus sequences using Geneious Prime v2025.1.2 (https://www.geneious.com).

For phylogenetic analysis, we used 8 newly generated 18S rDNA sequences and 36 additional capillariid sequences retrieved from GenBank. *Trichuris suis* (Schrank, 1788) was selected as the outgroup following previous studies (Sakaguchi et al., 2020; Svitin et al., 2021). All sequences were aligned and trimmed to the length of the shortest sequence using MEGA 12 (Kumar et al., 2024). The GTR+G+I model was identified in MEGA 12 as the best-fitting nucleotide substitution model and used for both Bayesian inference (BI) and Maximum Likelihood (ML) analyses. BI analysis was performed in MrBayes v3.2.2 using four Markov chains for 10,000,000 generations, sampling every 1000 generations, with the first 25% of samples discarded as “burn-in”. ML analysis was conducted in MEGA 12 with nodal support estimated from 500 bootstrap pseudo-replications. The resultant tree was visualised using FigTree v1.4.3.

## 3 Results

In total, 14 specimens of *A. tritonispunctati* were recovered from the intestines of newts, but only two males and two adult females were intact enough for complete morphometric measurements. A third male had a damaged anterior end, though the bursa and spicule were suitable for measurements. Due to the limited number of intact specimens, the morphological description was based on two males and two females, and the measurements for the posterior region in males were obtained from three specimens.

### 3.1 *Amphibiocapillaria tritonispunctati* (Diesing, 1851)

#### 3.1.1 Taxonomic summary

*Host*: *Triturus dobrogicus* (Caudata: Salamandridae), Danube crested newt.

*Locality*: Tuzlivski Lymany (45.6706, 29.8818), National Nature Park, Odesa Region, Ukraine.

*Site in host*: Intestine.

*Voucher material*: Voucher specimens are deposited in the collection of the Department of Parasitology of the I. I. Schmalhausen Institute of Zoology, NAS of Ukraine, under the accession numbers: NAMT1.1. IZSHK, NAMT2.1. IZSHK and NAMT3.1. IZSHK.

*Representative DNA sequences*: Sequence data were deposited in the GenBank database under the accession numbers PX249694 (*cox*1) and PX263206 (18S rRNA gene).

#### 3.1.2 Description

**Fig. 1:**
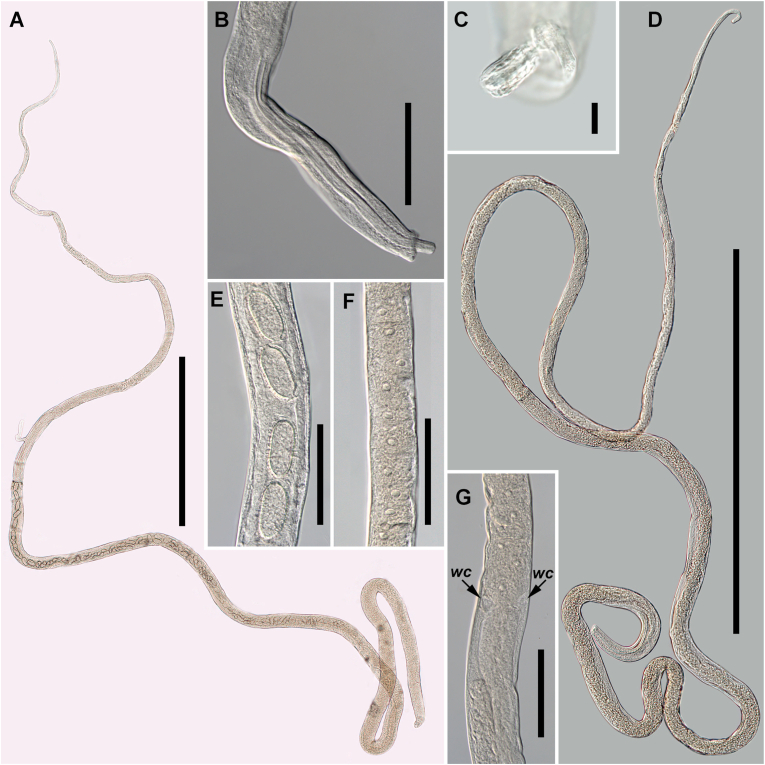
Photomicrographs of *Amphibiocapillaria tritonispunctati* from *Triturus dobrogicus*. **A** Female, total view. **B** Posterior region of male, lateral view. **C** Ornamentation on the protruding spicular tip. **D** Male, total view. **E** Fragment of body at uterus region, lateral view. **F** Fragment of body at level near stichosome mid-length, female, lateral view. **G** Fragment of body at level of stichosome posterior end, female, lateral view. *Abbreviation*: *wc*, wing-like cells. *Scale bars*: **A**, 1 mm; **B**, **E**, **F**, **G**, 100 μm; **C**, 10 μm; **D**, 500 μm.

*General* [Based on four specimens; Fig. 1.] Body long, filiform, narrow anteriorly, widening posteriorly (Fig. 1A–D). Both body extremities rounded. Bacillary bands narrow, nearly indistinct, extending almost along entire body length. Muscular part of oesophagus short, narrow in anterior part, evenly widening towards posterior extremity. Stichosome long, comprising of short stichocytes with large, distinct nuclei (Fig. 1F). Wing-like cells prominent, located at stichosome end (Fig. 1G). Position of nerve-ring varying within anterior third of muscular part of oesophagus.

*Male* [Morphometric data based on 2 intact adult specimens and fragments of 1 specimen.] Body 5.7–7.0 mm long, with maximum width of 59–64 (Fig. 1D). Lateral bacillary bands narrow, 5–7 wide at level of muscular oesophagus end. Oesophagus 3.2–3.7 mm long, comprising 53.8–56.6% of body length. Muscular part of oesophagus 344–402 long, comprising 5.8–6.0% of body length or 10.7% (in both specimens) of total oesophagus length. Stichosome 2.9–3.3 mm long, with 82–128 stichocytes. Nerve-ring encircling muscular part of oesophagus, 97–124 from anterior extremity of body or 24.1–36.0% of muscular oesophagus length. Spicular sheath with fine striations composed of minute flat spikes clearly visible at high magnifications (Fig. 1B and C). Spicule distinct, well-sclerotised with rounded tip, 216, 259, and 279 long and 5, 6, and 7 wide. Bursa narrow, slightly elevated, consisting of 2 lobes, each bearing small papilla near cloacal opening. Bursa 23, 23, and 28 long. Tail short, rounded 13, 13, and 15 long.

*Female* [Morphometric data based on 2 adult specimens.] Body 9.2–9.3 mm long, with maximum width of 73–87. Bacillary bands 9–10 wide at level of muscular oesophagus end. Oesophagus 3.3–3.6 mm long, comprising 35.3–39.4% of body length. Muscular part of oesophagus 369–400 long comprising 4.0–4.3% of body length or 10.2–12.2% of total oesophagus length. Stichosome 2.9–3.3 mm long, with 128–136 stichocytes. Nerve-ring encircling muscular part of oesophagus, 137–151 from anterior extremity of body or 37.1–37.8% of muscular oesophagus length. Vulva prominent, with slightly elevated anterior lip, located at level of stichosome posterior end. Vagina short, muscular. Eggs numerous (80–116), occupying most of uterine lumen, elongated, without protruding polar plugs, 52–60 (mean 55, *n* = 10) long and 24–28 (mean 26, *n* = 10) wide (Fig. 1E). Eggs located close to vagina at morula stage while further ones on different stages of cleavage or uncleaved. Posterior extremity of body rounded, rectum straight, well-sclerotised, anus terminal.

#### 3.1.3 Remarks

The species was formally described by Diesing (1851) based on references to findings of capillariid nematodes from *Triturus* spp. in earlier works by Dujardin, with the first report (Dujardin, 1843) from *Triturus cristatus* (Laurenti) in France. The species was subsequently reported in various caudate amphibians throughout Europe, Asia and North America (Moravec and Lomakin, 1982; Moravec, 1986, 2001; Joy and Scott, 1997). In Ukraine, Ryzhikov et al. (1980) first recorded *A. tritonispunctati* from *T*. *cristatus* and *Ichthyosaura alpestris* (Laurenti) under the name *Thominx filiformis*. Later, Moravec and Lomakin (1982) identified *A. tritonispunctati* from *T*. *cristatus* in the Danube Delta. The Danube crested newt, *T. dobrogicus*, was initially considered a subspecies of *T. cristatus*. After it was elevated to species status, Pysanets (2007) showed that the Danube Delta population is predominantly *T. dobrogicus*. Therefore, it is highly likely that the specimens studied by Moravec and Lomakin (1982) were in fact from *T. dobrogicus*. Our material consisted of newts likely washed ashore from the lower Dnipro River. Therefore, we consider them to originate from the somewhat isolated population of *T. dobrogicus* previously described there by Pysanets (2007). Given the wide host range reported for *A. tritonispunctati* (including newts from Ukraine) and its key morphological features (presence or absence of spicules, structure and ornamentation of specular sheathes, number and arrangement of stichocytes, and morphology, size, and number of eggs), we identified the present capillariid specimens as *A. tritonispunctati*. The morphometric data also correspond to previous species descriptions based on material from newts collected in Chechia and Poland (Moravec and Lomakin, 1982; Bertman and Okulewicz, 1987).

### 3.2 Molecular analysis

**Fig. 2:**
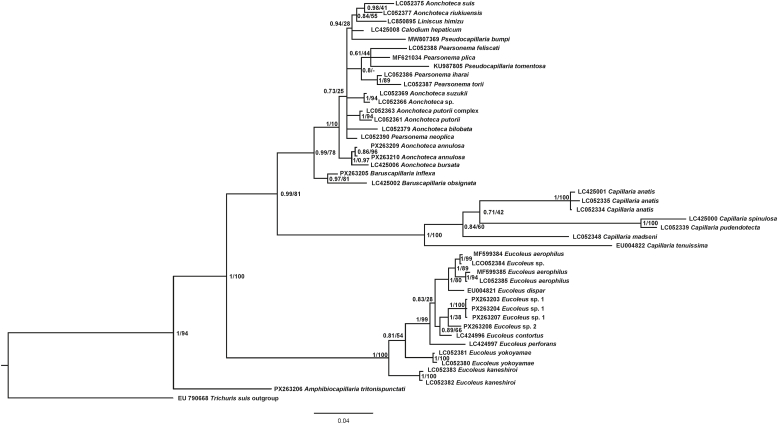
Bayesian inference phylogenetic tree for 44 species of the Capillariidae based on 1525 nucleotide-long fragments of the 18S rRNA gene. Nodal support is shown for both BI and ML as BI/ML. The scale bar indicates the number of substitutions per site.

In total, we generated eight new 18S rDNA sequences and one *cox*1 sequence to facilitate DNA barcoding (Table 1). In addition to *A*. *tritonispunctati*, these included four other species, *A. annulosa*, *B. inflexa*, *Eucoleus* sp. 1, and *Eucoleus* sp. 2. The eight newly generated sequences, together with 37 sequences retrieved from GenBank, were used for phylogenetic reconstruction. Both BI and ML analyses produced virtually identical topologies with comparable nodal support, so we present the BI tree with indication of the branch bootstrap support values from ML. In this phylogeny (Fig. 2), *A. tritonispunctati* was recovered at the base of the Сapillariidae clade with 100% posterior probability and 96% bootstrap support, suggesting it is the earliest-diverging lineage. *Eucoleus* spp. formed a well-supported monophyletic clade. *Capillaria* spp. from various birds were clustered into a robustly supported clade, which stands apart from the rest by a clear genetic distance. The last major clade comprises species from several genera, with *Baruscapillaria inflexa* and *B. obsignata* forming a sister group to the rest of the clade, which is composed of 14 sequences for species of the genera *Pseudocapillaria*, *Aonchotheca*, *Capillaria*, and *Pearsonema* found in birds, mammals, and fishes. Notably, our sequences of *A. annulosa* from a rodent (bank vole) formed a well-supported lineage with those of *Aonchotheca bursata* (Freitas & Almeida, 1934) from a bird (common pheasant).

**Table 1:** Details of the newly generated 18S ribosomal RNA gene and cytochrome *c* oxidase subunit 1 gene (*cox*1) sequences for capillariids.

Species	Host	Country	Coordinates	Collection date	Gene	GenBank ID
*Aonchotheca annulosa*	*Myodes glareolus*	Slovakia	48.7855, 21.2044	July 2023	18S rRNA	PX263209
*Aonchotheca annulosa*	*Myodes glareolus*	Slovakia	48.7514, 21.3437	July 2024	18S rRNA	PX263210
*Amphibiocapillaria tritonispunctati*	*Triturus dobrogicus*	Ukraine	45.6706, 29.8818	June 2023	18S rRNA	PX263206
*Amphibiocapillaria tritonispunctati*	*Triturus dobrogicus*	Ukraine	45.6706, 29.8818	June 2023	*cox*1	PX249694
*Baruscapillaria inflexa*	*Sturnus vulagaris*	Ukraine	50.5791, 30.2151	June 2017	18S rRNA	PX263205
*Eucoleus* sp. 1	*Sturnus vulagaris*	Ukraine	50.5791, 30.2151	June 2017	18S rRNA	PX263203
*Eucoleus* sp. 1	*Sturnus vulagaris*	Ukraine	50.5791, 30.2151	June 2017	18S rRNA	PX263204
*Eucoleus* sp. 1	*Sturnus vulagaris*	Ukraine	50.5791, 30.2151	June 2017	18S rRNA	PX263207
*Eucoleus* sp. 2	*Chroicocephalus ridibundus*	Ukraine	51.8139, 31.2357	August 2016	18S rRNA	PX263208

## 4 Discussion

The systematics of nematodes is often complicated by a lack of prominent morphological characters and by scarce molecular data. The application of modern molecular techniques helped illuminate the validity of several nematode taxa; however, many issues remain unresolved (Nisa et al., 2022). Among parasitic nematodes, the Capillariidae remains the most taxonomically contentious family, with recognised genera ranging from one to more than 20, depending on the authors’ interpretations of morphological characters (Yamaguti, 1961; Moravec, 1982, 2001). Moravec (1982, 2001) proposed a system of the Capillariidae primarily based on the morphology of the male caudal extremity (papillae, various cuticular lobes and membranes, structure and ornamentation of the spicule, etc.), which became the most accepted by different authors (Gibbons, 2010; Tamaru et al., 2025).

Subsequent phylogenetic analyses based on 18S rDNA and mitochondrial genome sequences confirmed the validity of some genera. All previous studies based on 18S rDNA sequences illustrated two well-supported monophyletic clades consisting of *Capillaria* spp. and *Eucoleus* spp. (Tamaru et al., 2015, 2025; Borba et al., 2019; Sakaguchi et al., 2020; Svitin et al., 2021; Deng et al., 2021). Comparing the mitochondrial genomes also confirmed the distinctness of *Capillaria* and *Eucoleus*, in addition to two other genera, *Pseudocapillaria* and *Aonchotheca* (see Deng et al., 2021). The present study confirmed those results, maintaining a well-supported separation of the *Eucoleus* clade after the inclusion of our new sequences. Adding *B. inflexa* to the phylogenetic analysis also confirmed the generic status of *Baruscapillaria* as it formed a well-supported clade with *B. obsignata* distinct from other genera in the clade.

At the same time, adding sequences of *A. annulosa* did not resolve the situation with the genus. The newly generated sequences of *A. annulosa* recovered from bank voles grouped with *A. bursata* from pheasants, whereas the three other species of *Aonchotheca* from mammals formed either a basal branch or a weakly supported polytomy. Lastly, as in the previous study by Borba et al. (2019), *A. riukiuensis* (Shoho & Machida, 1979) clustered with *Aonchotheca suis* (Yamaguti, 1943). It should be noted that both species were recovered from the same host species, the wild boar. Similar to prior work, species of *Pearsonema* clustered together, whereas the two species of *Pseudocapillaria* did not share a common ancestor (Borba et al., 2019; Sakaguchi et al., 2020; Svitin et al., 2021). The sole representative of *Amphibiocapillaria* appeared in the basal position relative to all other capillariids with strong nodal support. Such divergence may justify elevating the genus to subfamily status; however, we refrain from proposing this until data from more species of the family are available.

It is worth noting that *A. tritonispunctati* is one of the most widespread capillariids in European newts and the only currently valid species that parasitises the intestine of European salamanders. However, such a broad host range of the nematode may indicate that this nominal species actually comprises multiple genetic lineages, potentially forming a cryptic species complex. Therefore, although we are confident in identifying our specimens as *A. tritonispunctati*, we supplement the sequence data with detailed morphological descriptions in case future work reveals a cryptic species complex. In addition, we compared our sequences against GenBank records, but entries explicitly annotated as *Amphibiocapillaria* or *A. tritonispunctati* were few and comprised relatively short fragments, preventing their inclusion in the phylogenetic reconstruction. The only 18S rRNA gene record attributed to *A. tritonispunctati* from an *Andrias* sp. collected in Japan (GenBank: LC605543), reported by Tsuchida et al. (2021), overlaps our 18S rDNA sequence by 669 bp and shows 99.3% pairwise identity, which likely indicates that our material and LC605543 belong to the same species. We also found five *cox*1 records annotated in GenBank as *Capillaria* (*Amphibiocapillaria*) sp. from *Rana pirica* Matsui and *Hynobius retardatus* Dunn collected in Japan (GenBank: LC201947-LC201951); these *cox*1 fragments are 99.4–99.8% identical to one another. Their overlap with our *cox*1 sequence is only 74 bp (83.8–85.1% identity), which is too short for a reliable comparison; the low identity suggests they may not be the same species as our material, but longer sequences are needed to confirm this. These *cox*1 records were submitted to GenBank by Nakao in 2016, and we did not find an associated peer-reviewed publication, precluding morphological comparison with our specimens.

Our 18S rDNA sequence of *Eucoleus* sp. 2 from the black-headed gull differs by 1.6% from a GenBank record annotated as *Eucoleus contortus* (Creplin, 1839) (GenBank: LC424996), obtained from a domestic goose in Indonesia (Sakaguchi et al., 2020). The three *Eucoleus* sp. 1 sequences we obtained for the nematodes in the common starlings diverge from LC424996 by 3.1% and from *Eucoleus* sp. 2 by 3.2%. Because interspecific divergence at the nematode 18S rDNA locus is typically considered to mark separate species at around 1% (Porazinska et al., 2010; Tamaru et al., 2025) and given that our four sequences obtained from two hosts (the black-headed gull and the common starling), together with the GenBank sequence from the domestic goose, cluster within the same clade, these values of divergence point to three closely related but distinct genetic lineages. Morphological identification of the *Eucoleus* spp. specimens to the species level was not possible because only females were available for examination. *Eucoleus contortus* is widely distributed and infects multiple avian orders, including Anseriformes, Charadriiformes, Galliformes, Falconiformes, and Passeriformes. In the Palaearctic, it is the only *Eucoleus* species reported from hosts of the Laridae (Baruš et al., 1978). Importantly, Baruš et al. (1978) reviewed an earlier hypothesis that *E. contortus* should be divided into separate species according to their relationships with the systematic groups of definitive hosts, but rejected it due to insufficient morphological evidence and synonymised those taxa under *E. contortus*. Our results indicate that this earlier hypothesis deserves revisiting. Pending additional new evidence, we therefore regard the lineages detected here as plausible species of an *E. contortus* complex.

Both of our sequences of *A. annulosa* were 100% identical to the GenBank record KY488185, which is also annotated as *A. annulosa* recovered from the bank vole in Poland (Buńkowska-Gawlik et al., 2017). Before DNA extraction, we identified a male specimen morphologically and, following the criteria of Behnke and Jackson (2024), namely, that *Aonchotheca* from European rodents with a spicule longer than 0.9 mm should be assigned to *A. annulosa*. Our findings thus provide additional evidence that specimens of *Aonchotheca* with a long spicule, found in European rodents, belong to a single species, *A. annulosa*.

## 5 Conclusions

The study showed that *Amphibiocapillaria tritonispunctati* exhibits significant genetic divergence from other family members. This may suggest raising the genus *Amphibiocapillaria* to a higher taxonomic rank when more data are available. The findings also confirmed *Eucoleus*, *Capillaria*, and *Baruscapillaria* as monophyletic groups. The phylogenetic position of *Aonchotheca* and *Pseudocapillaria* remains unclear and requires more research. The results further imply the existence of a species complex within *Eucoleus contortus*. In summary, each new sequence or morphological insight is another piece of the puzzle. Only by gradually assembling these pieces can we reveal the taxonomy, evolution, host specificity, and distribution of the Capillariidae.

## Ethical approval

All nematode specimens originated from the parasitological collection of the I. I. Schmalhausen Institute of Zoology, NAS of Ukraine, and no hosts were deliberately euthanised for the present study. The rodents from Slovakia were found dead, and the carcasses had been handled following authorisation by the Ministry of Environment of the Slovak Republic under permit No. 498/2018-6.3. All applicable institutional, national, and international guidelines were followed during all procedures.

## CRediT authorship contribution statement

**Roman Svitin:** Conceptualisation, Formal analysis, Investigation, Writing – original draft, Writing – review & editing. **Yaroslav Syrota:** Investigation, Resources, Writing – review & editing. **Yuriy Kuzmin:** Writing – review & editing, Investigation. **Valeriia Dupak:** Writing – review & editing, Investigation. **Oksana Nekrasova:** Writing – review & editing, Resources. **Oleksii Marushchak:** Writing – review & editing, Resources. **Kateryna Antipova:** Writing – review & editing, Investigation. **Oksana Greben:** Writing – review & editing, Investigation. **Zuzana Hurníková:** Writing – review & editing, Resources. **Nataliia Brusentsova:** Writing – review & editing, Resources. **Martina Miterpáková:** Writing – review & editing, Resources.

## Statement on the use of AI-assisted technologies

The authors used Grammarly (https://www.grammarly.com/) to correct grammar and improve readability; all edits were reviewed by the authors, who take full responsibility for the final content.

## Funding

The study was financially partially supported by the 10.13039/100018227National Research Foundation of Ukraine (project No. 2023.03/0068 to Roman Svitin, Valeria Dupak, Oleksii Marushchak, Kateryna Antipova, and Oksana Greben), the Slovak Research and Development under contract No. APVV-21-0166 (Martina Miterpáková, Zuzana Hurníková, and Yaroslav Syrota) and Tubitak-2024-01 (Martina Miterpáková, Zuzana Hurníková), and NextGenerationEU through the Recovery and Resilience Plan for Slovakia under project No. 09I03-03-V01-00046 (Yaroslav Syrota).

## Declaration of competing interests

The authors declare that they have no known competing financial interests or personal relationships that could have influenced the outcomes of this study.

## Data Availability

The data supporting the conclusions of this article are included in this published article. The newly generated sequences were submitted to the GenBank database under the accession numbers PX263209 and PX263210 (*Aonchotheca annulosa*), PX263206 and PX249694 (*Amphibiocapillaria tritonispunctati*), PX263205 (*Baruscapillaria inflexa*), PX263203, PX263204 and PX263207 (*Eucoleus* sp. 1), and PX263208 (*Eucoleus* sp. 2).
